# Evaluation of the telehealth making sense of brain tumor psychological support intervention for people with primary brain tumor and their caregivers: A randomized controlled trial

**DOI:** 10.1002/pon.6189

**Published:** 2023-07-06

**Authors:** Tamara Ownsworth, Suzanne Chambers, Stephanie Jones, Giverny Parker, Joanne F. Aitken, Matthew Foote, Louisa G. Gordon, David H. K. Shum, Julia Robertson, Elizabeth Conlon, Mark B. Pinkham

**Affiliations:** ^1^ School of Applied Psychology Griffith University Brisbane Queensland Australia; ^2^ The Hopkins Centre Menzies Health Institute of Queensland Griffith University Brisbane Queensland Australia; ^3^ Faculty of Health Sciences Australian Catholic University Brisbane Australia; ^4^ Cancer Council Queensland Brisbane Queensland Australia; ^5^ Department of Radiation Oncology Princess Alexandra Hospital Brisbane Queensland Australia; ^6^ School of Medicine University of Queensland Brisbane Queensland Australia; ^7^ QIMR Berghofer Medical Research Institute Brisbane Queensland Australia; ^8^ Department of Rehabilitation Sciences The Hong Kong Polytechnic University Hong Kong China; ^9^ Summer Foundation Ltd Melbourne Victoria Australia

**Keywords:** caregivers, intervention, primary brain tumor, psychological support, randomized controlled trial, telehealth

## Abstract

**Objective:**

This pragmatic randomized control trial aimed to evaluate clinical efficacy of the Making Sense of Brain Tumour program delivered via videoconferencing (Tele‐MAST) for improving mental health and quality of life (QoL) relative to standard care in individuals with primary brain tumor (PBT).

**Method:**

Adults with PBT experiencing at least mild distress (Distress Thermometer ≥4) and caregivers were randomly allocated to the 10‐session Tele‐MAST program or standard care. Mental health and QoL were assessed pre‐intervention, post‐intervention (primary endpoint), and 6‐weeks and 6‐months follow‐up. The primary outcome was clinician‐rated depressive symptoms on the Montgomery‐Asberg Depression Rating Scale.

**Results:**

82 participants with PBT (34% benign, 20% lower‐grade glioma, 46% high‐grade glioma) and 36 caregivers were recruited (2018–2021). Controlling for baseline functioning, Tele‐MAST participants with PBT had lower depressive symptoms at post‐intervention (95% CI: 10.2–14.6, vs. 15.2–19.6, *p* = 0.002) and 6‐weeks post‐intervention (95% CI: 11.5–15.8 vs. 15.6–19.9, *p* = 0.010) than standard care, and were almost 4 times more likely to experience clinically reduced depression (OR, 3.89; 95% CI: 1.5–9.9). Tele‐MAST participants with PBT also reported significantly better global QoL, emotional QoL and lower anxiety at post‐intervention and 6‐weeks post‐intervention than standard care. There were no significant intervention effects for caregivers. At 6‐months follow‐up participants with PBT who received Tele‐MAST reported significantly better mental health and QoL relative to pre‐intervention.

**Conclusions:**

Tele‐MAST was found to be more effective for reducing depressive symptoms at post‐intervention than standard care for people with PBT but not caregivers. Tailored and extended psychological support may be beneficial for people with PBT.

## 1 INTRODUCTION

Primary brain tumors (PBT) pose a threat to life and result in diverse functional impairments that impact individuals' independence, social participation and quality of life (QoL).^1^ PBT broadly encompasses three subtypes: benign (non‐malignant) tumor, lower‐grade glioma (less aggressive initially, but risk of progression or recurrence over time) and high‐grade glioma (malignant). Despite variations in disease characteristics, treatment pathways and prognosis,^2^ individuals with PBT regardless of subtype experience stressors related to diagnosis and complex neurocognitive impairments which significantly impact their mental health.^2^, ^3^ High rates of depression and anxiety (30%–50%) persist beyond the initial treatment phase for both individuals with PBT and caregivers.^4^, ^5^

Despite the significant psychosocial impacts, there are few evidence‐based interventions for improving mental health and QoL of people with PBT and their caregivers.^6^, ^7^ In the first controlled psychosocial intervention trial for people with PBT, Ownsworth et al.^8^ evaluated the Making Sense of Brain Tumor (MAST) program, delivered face‐to‐face in people's homes (*n* = 50). Developed to address the psychological support needs of people with PBT, this 10‐session program was guided by the sense of coherence framework,^9^ aiming to increase individuals' understanding of their illness (comprehensibility), coping resources (manageability) and ability to find meaning in their life situation (meaningfulness). Sessions were tailored to allow a combination of individual and couple sessions as appropriate to the goals of the person with PBT. The MAST condition was associated with significantly greater improvements in mental health and QoL. Caregiver involvement was associated with lower depression for the person with PBT. At 6‐months follow‐up, participants with PBT reported significantly better mental health and QoL relative to pre‐intervention levels.^8^

The efficacy of the MAST program for managing depression has been recognised by international palliative care guidelines.^7^ However, face‐to‐face delivery in the home may not be feasible, and access to clinic‐based psychological support may be limited due to transport and geographic barriers, symptom burden and financial strain.^10^ Further, given the recent experience of lockdowns in the pandemic the need for remote delivery mechanisms to provide access to psychological care has escalated.

A systematic review^11^ of supportive care delivered via telehealth platforms identified that remote intervention delivery was generally feasible (*M* accrual = 68%; *M* adherence = 74%) and acceptable (*M* satisfied = 81%) for the PBT population. Adherence rates were higher and clinical gains were more evident for interventions involving interaction with clinicians as opposed to self‐guided interventions.^11^

Based on positive findings of the face‐to‐face MAST,^8^ and in line with the ORBIT model for behavioral treatment development,^12^ we initially piloted feasibility and acceptability of remote delivery of MAST (Tele‐MAST) via telephone^13^ and videoconferencing.^14^ In the videoconferencing pilot study,^14^ eight out of 10 individuals who commenced Tele‐MAST completed ≥8 sessions. Feedback highlighted the ease of access and benefits of remote delivery, tailored support and sense of connection with the therapist. Most participants (63%) demonstrated clinically meaningful improvement in mental health or QoL. The findings supported proceeding with a controlled trial of clinical efficacy.

### 1.1 Study objectives

The primary aim of this randomized controlled trial (RCT) was to evaluate the clinical efficacy of the Tele‐MAST intervention for improving mental health and QoL of adults with PBT relative to standard care.^15^ The trial was pragmatic in the sense that we aimed to determine whether an extended brain tumor‐specific intervention yields better clinical outcomes than existing care practices for people with PBT.^12^, ^15^

The hypotheses were:

1. At post‐intervention (primary endpoint) and 6‐weeks post‐intervention, Tele‐MAST participants with PBT would report significantly lower depressive symptoms than those receiving standard care after controlling for baseline functioning.
2. At post‐intervention and 6‐weeks post‐intervention, Tele‐MAST participants with PBT would report significantly lower anxiety and higher levels of QoL than those receiving standard care after controlling for baseline functioning.
3. Relative to pre‐intervention, participants with PBT would report significantly better mental health and QoL at 6‐month follow‐up after the Tele‐MAST intervention.

Further aims were to examine the impact of the Tele‐MAST program on caregivers' mood and QoL, identify factors related to intervention outcomes on the primary outcome (depressive symptoms), and examine the cost‐effectiveness of Tele‐MAST (reported separately).

## 2 METHOD

### 2.1 Study design and sample size

In this two‐arm pragmatic RCT, efficacy of the Tele‐MAST intervention was evaluated relative to standard care or existing cancer support services. Mental health and QoL were assessed at baseline (T1), immediately post‐intervention (T2), and 6‐weeks post‐intervention (T3), and 6‐months (T4) follow‐up post‐intervention. Standard care participants were offered Tele‐MAST after T3. The trial was prospectively registered with the Australian and New Zealand Clinical Trials Registry (ACTRN12618001737224) and reporting adhered to the protocol^15^ and CONSORT‐Outcomes 2022 extension.^16^

The previous RCT^8^ found moderate‐to‐large effect sizes for between‐group differences in mental health and QoL (*η*_ρ_^2^ = 0.12−0.17) for MAST, relative to wait list controls. An estimated moderate effect size (*η*_ρ_^2^ = 0.08) was used in a power analysis conducted through G*Power.^17^ With alpha set at 0.05, and power of 0.90, a sample size of *n* = 123 (62 per group) was required to detect a moderate‐sized difference in depressive symptoms between Tele‐MAST and standard care at T2 (primary endpoint) controlling for baseline functioning. Due to anticipated attrition (20%), the recruitment goal was *n* = 148.

### 2.2 Ethical approval

The research was approved by Human Research Ethics Committees (HREC) of Metro South Health (HREC/18/QPAH/95) and Griffith University (Ref: 2018/808).

### 2.3 Participants

Over a 3‐year period (November 2018–2021), participants with PBT were recruited from a community‐based cancer support service and metropolitan hospital in Brisbane, Australia. Participants were screened for eligibility by treating medical and nursing staff (hospital) and cancer support staff (community). They were eligible if they: (1) were aged ≥18 years; (2) had a benign or malignant PBT at any disease stage; (3) reported psychological distress (i.e., Distress Thermometer [DT] score ≥4)^18^; (4) displayed adequate cognitive capacity and English language skills; and (5) could reliably access the Internet and suitable electronic device (PC/laptop/tablet). Participants performing in the very impaired range (age‐adjusted Z‐score < −3) on a validated telephone‐based cognitive test^19^ and/or those with severe receptive and/or expressive aphasia as documented by referring professionals were excluded. Caregivers were eligible to participate if aged ≥18 years, had adequate English language skills and their relative with PBT had consented to participate.

### 2.4 Measures

The Brief Test of Adult Cognition by Telephone (BTACT)^19^ and Similarities subtest (Wechsler Adult Intelligence Scale‐Fourth edition^20^) were administered at baseline to assess participants' cognitive and language skills. Sociodemographic data were obtained via interview. Clinical data on tumor type and treatment were accessed from medical records.

#### 2.4.1 Clinical outcomes

Table S1 summarises outcome measures administered via telephone. Internal consistency of measures ranged from adequate to good (*α* = 0.74‐0.89). Outcome measures were administered by a researcher blinded to intervention allocation. The time interval between baseline and post‐intervention assessments was approximately 12–15 weeks for both conditions.

#### 2.4.2 Primary outcome

The Montgomery‐Asberg Depression Rating Scale (MADRS^21^), a clinician‐rated semi‐structured interview of depressive symptoms, was the primary measure of mental health. Assessors rate 10 items from 0 (no/minimal symptoms) to 6 (maximum symptoms), with total scores ≥12 signifying clinical levels of depression.^21^ The MADRS demonstrated good test‐retest reliability (*r* = 0.85) and sensitivity to change in the previous MAST study.^8^ As clinician‐rated and self‐report mood measures may yield different outcomes,^22^ a self‐report of depressive symptoms was also administered (see secondary outcomes). The minimal clinically important difference (MCID) of ≥6 for the MADRS^14^, ^15^ is consistent with 10% of the instrument's range. Fifty audiotaped interviews were assessed by two independent raters. Interrater reliability was excellent (*ICC* = 0.98) for the total score.^23^

#### 2.4.3 Secondary outcomes

Participants with PBT were also administered the DT,^18^ Functional Assessment of Cancer Therapy‐Brain (FACT‐Br),^24^ Depression subscale of the Depression Anxiety and Stress Scales‐21 (DASS‐21),^25^ Generalized Anxiety Disorder‐7 (GAD‐7),^26^ and McGill Quality of Life Questionnaire existential well‐being subscale (MQOL‐EW).^27^ Caregivers' mental health and QoL were assessed using DASS‐21 and WHO Quality of Life‐BREF (WHOQOL‐BREF).^28^

### 2.5 Procedure

#### 2.5.1 Participant consent and randomization

Following screening, potential participants with PBT were emailed information and consent forms and contacted via telephone by research personnel unfamiliar to participants to obtain informed consent and conduct the baseline assessment. Individuals with PBT discussed the study with caregivers who also provided informed consent via telephone.

Participants were randomized to the Tele‐MAST intervention or standard care by a researcher independent of the study. Randomization was stratified according to baseline distress (DT mild‐to‐moderate [4–7] vs. severe [≥8]) and family involvement (yes/no) in terms of whether caregivers consented to participate in the trial. A predetermined computer‐generated random sequence was used, with allocation concealed using sequentially numbered sealed opaque envelopes.

#### 2.5.2 Intervention procedures

Tele‐MAST participants received 10 × 1‐h sessions per week via Zoom videoconferencing from a psychologist previously unfamiliar to participants. Participants practiced receiving a call and navigating audio‐visual features on their device. As outlined in Table S2, based on the MAST therapy manual,^29^ psychologists delivered core sessions (1, 2 & 10) and tailored sessions (3–9) with modules selected based on participants' goals and caregivers' involvement (individual & couple sessions). Although modules selected and time allocated to each varied for tailored sessions, examples include psychoeducation on emotional and cognitive changes (1‐2 sessions), psychotherapy to address low mood and anxiety (4‐5 sessions) and strategy training to manage subjective cognitive effects (1‐2 sessions). Therapy sessions were audio‐recorded with a random selection (17%) reviewed to examine adherence to Tele‐MAST therapy protocol.

In terms of the control condition, standard psychosocial care for people with PBT varies across Australia.^10^ For people with cancer in the study context (Queensland) standard care is based on a stepped‐care model^30^ and individuals reporting at least mild distress (DT ≥ 4) can receive up to five telephone‐based sessions with a psychologist. Accordingly, all standard care participants were offered up to five fortnightly sessions of telephone‐based individual and/or couples therapy focusing on stressors and illness‐related concerns. In each condition, the Session Rating Scale (SRS)^31^ assessed participants' perception of the therapeutic relationship after every session, with average ratings (0 = least positive, 10 = most positive) calculated.

### 2.6 Data analysis

Data were screened for missingness and assumptions of parametric analyses were examined. Participants were included in analyses according to intervention allocation. A mixed‐model approach was employed with group allocation as the between‐subjects factor, time (post‐intervention, 6‐weeks post‐intervention) as the repeated factor and baseline functioning (T1) as the covariate to evaluate whether Tele‐MAST was more effective than standard care for primary and secondary outcomes at T2, and whether these effects were sustained at T3. Demographic or illness variables significantly associated with outcomes at T2 or T3 were included as covariates. Based on Little's test, data were missing completely at random, ᵡ^2^(*df* = 64, *N* = 80) = 52.76, *p* = 0.841. Missing data were estimated through multiple imputation with 20 imputed data sets.^32^ Data for two Tele‐MAST participants deceased before T2 were not imputed based on recommendations by Herbert et al.^33^

To investigate factors influencing intervention outcomes at T2 and T3, participants meeting the MCID on MADRS (‘improvers’) were identified. Demographic, illness and therapy‐related variables related to MCID outcomes were examined using *t*‐tests and chi‐square tests.^1^ Longer‐term outcomes for all participants receiving Tele‐MAST (immediately or after standard care) were examined at T4 relative to baseline.

## 3 RESULTS

### 3.1 Sample characteristics

**FIGURE 1:**
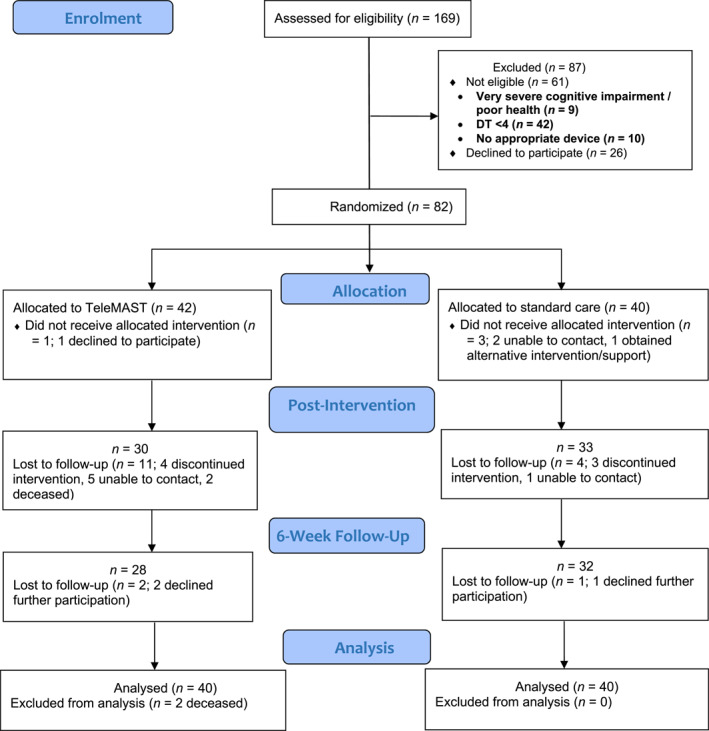
CONSORT Diagram for the Tele‐MAST clinical trial.

Between November 2018 and 2021, 82 people with PBT were recruited. During this time, 169 individuals were screened; 61 were ineligible (DT < 4) and 26 declined (see Figure 1). Most participants were female (61%), with mean age of 47.9 years (*SD* = 14.5) and time since diagnosis of 43.9 months (*SD* = 56.3). The most common PBT types were glioblastoma (27%), meningioma (21%) and oligodendroglioma (12%). Thirty‐six caregivers also participated; 64% female, mean age 46.1 (*SD* = 11.32), and were mainly spouses (81%).

Forty‐two participants were allocated to Tele‐MAST and 40 participants to standard care. There were no significant between‐group differences in demographic or illness‐related characteristics (see Table 1). Sixty‐three participants (77%) were retained at T2 and 60 (73%) were retained at T3 (see Figure 1). Forty‐two participants (51%) completed the T4 assessment 6‐months after Tele‐MAST. Attrition was higher in Tele‐MAST at T2 (29%) than standard care (18%). Results are based on the overall pooled imputed dataset (*n* = 80).

**TABLE 1:** Participant demographic and illness characteristics.

Characteristics	All (*n* = 82)	Tele‐MAST	Standard care	*ꭓ* ^2^/*t*
	*M* (SD), range/	(*n* = 42)	(*n* = 40)	
	*N*(%)	*M*(*SD*), range/*N*(%)	*M*(*SD*), range/*N*(%)	
Age (years)	47.90 (14.47),	48.89 (13.5),	46.88 (15.5)	0.34
	18–82	22–76	18–82	
Education (years)	14.17 (2.9),	14.14 (2.3),	14.2 (3.5),	0.08
	7–25	9–14	7–25	
Gender				
Female	50 (61)	23 (55)	27 (68)	1.32
Male	32 (39)	19 (45)	13 (32)	
Relationship status				
Single	25 (31)	12 (29)	13 (32)	0.55
In relationship	57 (69)	30 (71)	27 (68)	
Time since diagnosis (months)	43.93 (56.27),	37.6 (40.4),	50.6 (69.1),	0.90
	1–287	1–144	1–287	
Disease status				
Initial	66 (81)	35 (83)	31 (78)	0.31
Recurrence	16 (19)	7 (17)	9 (22)	
Tumor type				
Benign	28 (34)	15 (36)	13 (32)	0.84
Lower‐grade	16 (20)	9 (21)	7 (18)	
High‐grade	38 (46)	18 (43)	20 (50)	
Tumor grade				
I	20 (24)	10 (24)	10 (25)	2.38
II	24 (29)	14 (33)	10 (25)	
III	15 (18)	4 (12)	10 (25)	
IV	23 (28)	13 (31)	10 (25)	
Global cognitive status (BTACT)	−0.43 (0.90),	−0.44 (0.85),	−0.41 (0.97),	0.12
	−2.76‐1.73	−2.13‐1.73	−2.76‐1.29	
Verbal reasoning (WAIS‐IV similarities)	9.36 (2.84),	9.6 (2.62)	9.13 (3.10)	0.75
	1–14	1–14	1–14	

Abbreviations: BTACT, Brief Test of Adult Cognition by Telephone; WAIS, Wechsler Adult Intelligence Scale IV (4th edition).

### 3.2 Therapy sessions, alliance and adherence

Tele‐MAST participants completed on average 8.50/10 (*SD* = 2.7) sessions, whereas standard care participants completed 3.82/5 (*SD* = 1.7) sessions. The 18 caregivers in Tele‐MAST attended on average 1.25 (*SD* = 2.79, range 0–10) sessions, whereas none of the 18 caregivers in standard care participated in therapy. Therapy alliance according to SRS did not significantly differ between Tele‐MAST (*M* = 9.40, *SD* = 1.13) and standard care (*M* = 9.49, *SD* = 0.82, *t* = −0.35, *p* = 0.719).

Ratings of therapist adherence identified a high level of adherence (88%–100%) to Tele‐MAST components across sessions 1–9. Adherence was more variable (71%–100%) for therapists' exploration of personal gains in session 10.

### 3.3 Primary outcome

Baseline depressive symptoms on MADRS were comparable (*t* = −1.16, *p* = 0.252) between Tele‐MAST (*M* = 18.88 *SD* = 7.50) and standard care (*M* = 20.88, *SD* = 7.98) conditions. Controlling for baseline depressive symptoms and months post‐diagnosis, there was a significant intervention effect, *F* = 11.61, *p* = 0.001 (see Table S3). Pairwise comparisons indicated that Tele‐MAST participants demonstrated significantly lower depressive symptoms at T2 (*M* = 12.38, 95% CI: 10.2–14.6, *F* = 10.31, *p* = 0.002) and T3 (*M* = 13.66, 95% CI: 11.5–15.8, *F* = 6.78, *p* = 0.010) than standard care participants (T2: *M* = 17.43, 95% CI: 15.2–19.6; T3 *M* = 17.75, 95% CI: 15.6–19.9). Effect sizes were in the medium range (*η*_*p*_^2^ = 0.08–0.12). A significantly higher proportion of Tele‐MAST participants (70%) met the MCID (≥6) between baseline and T2 as compared to standard care (38%, *ꭓ* ^2^ = 8.50, *p* = 0.004; *φ*_*c*_ = 0.33; OR = 3.89, 95% CI: 1.5–9.9). A higher proportion of Tele‐MAST participants (58%) also met the MCID between baseline and T3 as compared to standard care; however, this was not significant (38%, *ꭓ* ^2^ = 3.21, *p* = 0.07; *φ*_*c*_ = 0.20; OR: 2.26, 95% CI: 0.92–5.5). Six Tele‐MAST and eight standard care participants experienced clinical deterioration (MADRS increased ≥6). No pattern was evident regarding illness characteristics for those who deteriorated; 4/6 in Tele‐MAST attended 3‐8 sessions and 2/6 attended 10, whereas 4/8 standard care participants attended 1‐3 sessions and 4/8 attended 4‐5.

### 3.4 Secondary outcomes

There were no significant baseline differences for secondary outcomes (See Table S3). Controlling for baseline functioning and relevant covariates, there was a significant intervention effect for DT (*F* = 4.43, *p* = 0.039), DASS‐depression (*F* = 11.67, *p* = 0.001), GAD‐7 (*F* = 8.56, *p* = 0.005), FACT‐G (*F* = 6.10, *p* = 0.016), FACT‐Physical (*F* = 5.30, *p* = 0.024), FACT‐Emotional (*F* = 10.83, *p* = 0.002), FACT‐Functional (*F* = 6.60, *p* = 0.012) and MQOL‐Existential (*F* = 5.71, *p* = 0.019). There was no significant intervention effect for social QoL (FACT‐Social: *F* = 0.50, *p* = 0.484) or self‐reported disease symptoms (FACT‐Br: *F* = 2.19, *p* = 0.143).

Pairwise comparisons showed that depression and anxiety levels were significantly lower and global QoL, emotional QoL and functional QoL were significantly higher for Tele‐MAST at both T2 and T3 compared to standard care (*η*_*p*_^2^ = 0.05–0.12; see Table S3). Although distress and physical QoL did not significantly differ between conditions at T2 (DT: *F* = 0.94, *p* = 0.33; *η*_*p*_^2^ = 0.01; FACT‐Physical: *F* = 2.12, *p* = 0.150; *η*_*p*_^2^ = 0.03), Tele‐MAST participants reported significantly lower distress and better physical QoL at T3 than standard care participants (DT: *F* = 6.15, *p* = 0.014; *η*_*p*_^2^ = 0.07; FACT‐Physical: *F* = 7.08, *p* = 0.009; *η*_*p*_^2^ = 0.08). Conversely, existential QoL was significantly higher at T2 for Tele‐MAST (*F* = 6.98, *p* = 0.01; *η*_*p*_^2^ = 0.08), but did not differ from standard care at T3 (*F* = 2.55, *p* = 0.114; *η*_*p*_^2^ = 0.03).

### 3.5 Factors related to intervention outcomes

Chi‐square tests and independent *t*‐tests identified that no demographic or clinical characteristics were significantly related to MCID outcomes on MADRS for Tele‐MAST or standard care (see Table S4).

### 3.6 Intervention outcomes for caregivers

As shown in Table S5, caregivers allocated to Tele‐MAST reported significantly lower depression and anxiety and higher psychological QoL at baseline than those allocated to standard care (*p* < 0.05). There were no significant between‐group differences in mental health or QoL across timepoints, controlling for baseline functioning.

### 3.7 Long‐term outcomes of Tele‐MAST program

Forty‐two participants with PBT underwent assessment at T4, including 19 participants who completed Tele‐MAST after initial allocation to standard care. Demographic and clinical characteristics did not significantly differ between participants completing Tele‐MAST and 6‐months follow‐up and those lost to follow‐up (*p* > 0.05). At T4, participants reported significantly lower depressive symptoms (*t* = 5.90, *p* < 0.001), distress (*t* = 5.84, *p* < 0.001), anxiety (*t* = 2.91, *p* = 0.006), and self‐reported disease symptoms (*t* = −2.35, *p* = 0.024), and better global (*t* = −3.81, *p* < 0.001), physical (*t* = −2.23, *p* = 0.032), emotional (*t* = −3.69, *p* < 0.001), functional (*t* = −4.94, *p* < 0.001) and existential (*t* = −3.55, *p* = 0.001) QoL, relative to T1 (Table S6). There were no significant differences in social QoL between T1 and T4 (*t* = −1.17, *p* = 0.251).

## 4 DISCUSSION

This pragmatic RCT evaluated clinical efficacy of the Tele‐MAST intervention relative to standard care for people with PBT. As hypothesized, Tele‐MAST participants reported significantly lower depressive (MADRS, DASS) and anxiety (GAD‐7) symptoms and better global, emotional and functional QoL at post‐intervention and 6‐weeks post‐intervention. Intervention outcomes were variable for distress (DT) and other QoL domains. There were no significant intervention effects for caregivers' mental health or QoL. At 6‐months follow‐up, participants completing Tele‐MAST had significantly better mental health and QoL compared to pre‐intervention levels.

The Tele‐MAST intervention was associated with significantly lower depressive symptoms at post‐intervention, which was sustained at 6‐weeks post‐intervention. Tele‐MAST participants were almost four times more likely (OR: 3.89) to demonstrate clinically meaningful change on MADRS at post‐intervention than standard care. Moreover, 79% of Tele‐MAST participants were in the clinical range for depression (MADRS ≥12) at baseline, whereas less than half were in this range at post‐intervention (43%) and 6‐weeks post‐intervention (48%). For standard care, the proportions were 88%, 75% and 78%, respectively. Notably, several participants experienced clinical deterioration on MADRS during Tele‐MAST (*n* = 6) and standard care (*n* = 8), although no pattern was evident regarding their illness characteristics.

Tele‐MAST was also associated with significantly lower anxiety and improved global, emotional and functional QoL relative to standard care, with effects sustained at 6‐weeks post‐intervention. Although distress scores (DT) did not differ at post‐intervention, Tele‐MAST participants reported lower distress at 6‐weeks post‐intervention than standard care. DT is a self‐reported distress rating selected due to its brevity (single‐item), whereas MADRS, a clinician‐rated measure of depressive symptoms, was selected as primary outcome because this 10‐item measure was considered more likely to be sensitive to intervention effects than the DT. Nonetheless, at 6‐weeks post‐intervention Tele‐MAST participants' distress levels were on average below clinical cut‐offs (DT < 4), whereas standard care participants' distress levels were above clinical cut‐offs. Hence, Tele‐MAST had enduring benefits for managing distress, which is a key focus of psycho‐oncology Clinical Practice Guidelines.^34^, ^35^ The current results compare favorably with outcomes of previous neuro‐oncology interventions,^6^, ^7^ including a 5‐week online self‐guided intervention for which no significant effects were found for mental health or QoL at post‐intervention or 12‐weeks post‐intervention.^36^

Tele‐MAST participants also reported better physical QoL at 6‐weeks post‐intervention relative to standard care, which may be due to improvements in mood affecting symptom experience (e.g., pain and energy). However, intervention effects were not sustained at 6‐weeks post‐intervention for existential QoL and did not extend to social QoL or self‐reported disease symptoms (e.g., sensory, motor & cognitive symptoms). The low involvement of caregivers may have impacted the meaningfulness component of Tele‐MAST beyond the intervention, given the influence of social support on meaning‐making.^37^

Although caregivers were encouraged to participate, less than half (43%) had family members involved and participation was often limited to 1‐2 sessions. In the face‐to‐face MAST,^8^ caregivers participated in 60% of programs with mean attendance of 5.4 sessions. It is possible the videoconferencing platform discouraged caregiver engagement, as opposed to therapists visiting in the home. Xiao et al.^38^ provided home‐based psychological care (*n* = 162) and reported reduced anxiety and depressive symptoms for individuals with PBT and caregivers relative to telephone review. However, due to non‐randomized allocation, these improvements may be due to other factors.

The lack of benefits of Tele‐MAST for caregivers' mental health and QoL may be partly due to their low uptake of sessions (*M* = 1.25, *SD* = 2.79) as well as not having inclusion criteria regarding their distress levels. Unexpectedly, caregivers receiving standard care reported greater depressive and anxiety symptoms at baseline than caregivers receiving Tele‐MAST. As the latter group were in the normal range for depressive, anxiety and stress symptoms (DASS), there was limited scope for gains in mental health. While sessions involving caregivers typically addressed shared goals (e.g., psychoeducation and communication skills), Tele‐MAST primarily focused on psychological well‐being of individuals with PBT, with sessions tailored according to their goals. Notably, participants with PBT were asked to discuss the study with caregivers to encourage their involvement. In future trials, it is recommended that researchers contact caregivers to clarify the scope for individual and couple therapy sessions. Previous interventions have typically addressed support needs of either people with PBT or caregivers.^14^ As an exception, Milbury et al.^39^ evaluated the efficacy of a couple‐based mindfulness meditation via FaceTime with 37 dyads. Although individuals with PBT reported significantly fewer disease symptoms, there were no significant improvements in caregivers' well‐being.

### 4.1 Study limitations

As a pragmatic trial, it was not possible to match therapy dosage between Tele‐MAST and standard care. Therapy emphasis also differed, with standard care focused mainly on stress management and coping^35^ whereas Tele‐MAST provided tailored psychoeducation regarding cognitive and emotional effects of brain tumor, compensatory strategy training, couple counselling and legacy projects. No caregivers elected to participate in standard care, and therefore caregiver involvement in therapy was not controlled for. Notably, ratings of therapeutic alliance did not significantly differ between intervention conditions. Hence, the current trial demonstrated that an extended brain‐tumor specific intervention yielded better clinical outcomes for people with PBT than brief telephone‐based counselling, the care standard for people with cancer experiencing distress in the study context.^40^

As another limitation, the target sample size (*n* = 148) was not achieved within the project timeframe and attrition was higher (27%) than expected at 6‐weeks post‐intervention, which may have affected statistical power for some analyses. The medium effect size (*η*_ρ_^2^ = 0.12) for differences in depressive symptoms at post‐intervention indicated the trial was adequately powered (>0.90) for the primary outcome. However, due to the modest sample size, multivariate analysis of factors associated with MCID on the MADRS was not feasible.

A strength of the current study was inclusion of the 6‐week follow‐up, demonstrating post‐intervention gains were largely sustained in the short‐term. Although the improved long‐term mental health and QoL outcomes at 6‐months relative to pre‐intervention are promising, these cannot be directly attributed to Tele‐MAST due to the study design and likely cohort biases. Participants retained at long‐term follow‐up are less likely to have experienced functional decline than those withdrawing for health reasons or unable to be contacted. Finally, participant and therapist blinding were not possible, increasing the potential for overestimated treatment effects.^40^

### 4.2 Clinical implications

Overall, the findings support the efficacy of a telehealth format of MAST for improving mental health and QoL of individuals with PBT. Informed by research on psychosocial support needs of people with PBT,^37^ the focus on sense of coherence and tailored therapy components may have enhanced participants' ability to manage psychological effects of their illness. However, caregiver engagement was lower than the face‐to‐face MAST^8^ and participant drop‐out was higher for Tele‐MAST than standard care, potentially due to the greater therapy time commitment. These findings highlight the need to explore individual and caregiver preferences regarding intervention format (face‐to‐face/online; individual/couple) and intensity with a view to delivering person‐centred programs in practice. Aligned with the protocol,^15^ we plan to examine cost‐effectiveness of Tele‐MAST relative to standard care. We are also currently trialling a caregiver specific Tele‐MAST program.

## 5 CONCLUSIONS

This pragmatic RCT supported the efficacy of Tele‐MAST for improving mental health and QoL in people with PBT. Research focused on support needs and intervention preferences of caregivers and understanding who most benefits from extended psychological support is recommended to support the translation of Tele‐MAST into wider practice.

## AUTHOR CONTRIBUTIONS

All authors contributed to the study conception and design. Data collection was performed by Stephanie Jones and Giverny Allen and data analysis was conducted by Elizabeth Conlon and Tamara Ownsworth. The first draft of the manuscript was written by Tamara Ownsworth and all authors commented on previous versions of the manuscript. All authors read and approved the final manuscript.

## CONFLICT OF INTEREST STATEMENT

The authors have no relevant financial or non‐financial interests to disclose.

## ETHICS STATEMENT

This study was performed in line with the principles of the Declaration of Helsinki. Approval was granted by the Ethics Committees of Metro South Health (Date: 20/9/2018; HREC/18/QPAH/95) and Griffith University (Date: 30/10/2018; GU Ref No: 2018/808).

## TRIALS REGISTRATION

The trial was prospectively registered with the Australian and New Zealand Clinical Trials Registry (ACTRN12618001737224).

## CONSENT TO PARTICIPATE

Informed consent was obtained from all individual participants included in the study.

## Supporting information

Supporting Information S1

## Data Availability

The datasets generated during and/or analysed during the current study are available from the corresponding author on reasonable request.
