# Supplementary material for: Evaluation of the telehealth making sense of brain tumor psychological support intervention for people with primary brain tumor and their caregivers: A randomized controlled trial
Source: Psychooncology. 2023 Jul 6;32(9):1385–94. doi: 10.1002/pon.6189 (PMC10946492; doi:10.1002/pon.6189)
Supplement: Supplementary file 1 — Supporting Information S1 [file PON-32-1385-s001.docx]

**SUPPLEMENTARY TABLES**

Table S1

*Summary of Clinical Outcome Measures and Cronbach’s Alpha for Current Sample*

| Measures | Items, construct and scoring | Cronbach’s alpha  (*n* = 82) |
| --- | --- | --- |
| Primary outcome |  |  |
| Montgomery-Asberg Depression Rating Scale | 10 clinician-rated items (0-6) assessing the presence and severity of depressive symptoms  Score range: 0-60, higher scores reflect more severe depression | .80 |
| Secondary outcomes |  |  |
| Distress Thermometer | Single item rating of psychological distress (0-10); >4 used to indicate distress warranting psychological support. Higher scores reflect greater distress. | NA |
| Functional Assessment of Cancer Therapy-Brain | General (FACT-G): 27 items assessing global QoL and physical, social, emotional, and functional QoL. Higher scores reflect better QoL.  Brain module (FACT-Br): 23 items assessing brain tumour specific symptoms^a^. Lower scores indicate greater **subjective disease symptoms.** | .84  .82 |
| Depression scale, DASS-21 | 7 items assessing symptoms of low mood (cognitive and affective symptoms of depression) | .89 |
| Generalized Anxiety Disorder-7 | 7 items assessing cognitive and affective components of anxiety | .88 |
| McGill Quality of Life- Existential well-being | 6 items assessing existential well-being (e.g., sense of control, purpose, meaning in existence & self-worth) | .87 |
|  |  |  |
| Family caregivers |  |  |
| DASS-21 | 21 items (7 items per subscale) assessing symptoms of depression, anxiety and stress. | .74-.80 |
| WHOQOL-BREF | 26 items assessing physical, psychological, social and environmental health and wellbeing (raw scores are transformed to a 0-100 scale, higher scores reflect better quality of life) | .78-.81 |

Table S2

*The Tele-MAST Program Components: Core and Tailored Sessions Guided by the Therapist’s Manual (Stewart & Ownsworth, 2014)*

| Session number | Program components: Session objectives and content |
| --- | --- |
| 1: Telling my story (core) | - Individuals share their personal experience of symptom onset, diagnosis, treatment and the everyday impact of the illness. |
| 2: Values and goal setting (core) | - Exploring the psychosocial effects of the illness (e.g., independence, work, relationships) - Individuals identify their current values and set goals for therapy, which guide the selection of therapy modules. |
| 3-9: Tailored psychotherapy and cognitive rehabilitation | **Start of session: asking participants about their week (e.g., health updates); revisiting goals and providing a rationale for the session focus; and introducing topic/approach of relevant module.**  Example modules include:   - Psychoeducation on the effects of brain tumour - Fatigue education and management - Education regarding emotional changes (low mood, anxiety, stress response, anger) - Education regarding cognitive effects (attention, memory, executive function) - **Training in compensatory strategies (e.g., memory, planning and organization aids)** - **Psychotherapy approaches to address low mood, anxiety, stress and anger issues.** - Behavior and interpersonal skills (e.g., motivation, apathy, assertiveness) - Relationship counselling (e.g., communication, coping, problem-solving, conflict resolution) - Addressing fears/concerns regarding end-of-life (existential approaches) - Creating legacy projects |
| 10: Program review and maintenance (core) | - Reviewing progress relevant to goals, the sense of coherence framework and planning for skills maintenance and upcoming stressors. |

Table S3

Mixed-model Analyses Comparing the Tele-MAST and Standard Care Groups at Post-Intervention and 6-Week Post-Intervention Follow-up Controlling for Baseline Functioning and Relevant Covariates

| Outcome measure | Baseline  *M*(*SD*) | Post-intervention  *M*(SD) | 6-weeks post-intervention  *M*(*SD*) | Covariates (in addition to baseline functioning) | Overall  *F* | Pairwise comparisons  T1-T2  *EMM (SE)* | **Effect size^a^ (η_p_^2^),**  95% CI | Pairwise comparisons  T1-T3  *EMM (SE)* | **Effect size (η_p_^2^),**  95% CI |
| --- | --- | --- | --- | --- | --- | --- | --- | --- | --- |
| MADRS | *t* = -1.16, *p* = .252 |  |  | Months post-diagnosis | *F* = 11.61** | *F* = 10.31** | **0.12** | *F* = 6.78* | **0.08** |
| Tele-MAST | 18.88 (7.50) | 11.87 (6.19) | 13.14 (6.38) |  |  | 12.38 (1.11) | 10.2-14.6 | 13.66 (1.10) | 11.5-15.8 |
| Standard care | 20.88 (7.98) | 17.94 (9.39) | 18.26 (9.05) |  |  | 17.43 (1.11) | 15.2-19.6 | 17.74 (1.10) | 15.6-19.9 |
|  |  |  |  |  |  |  |  |  |  |
| Distress (DT) | *t* = -0.55, *p* = .587 |  |  | Months post-diagnosis | *F* = 4.43* | *F* = 0.94 | **0.01** | *F* = 6.15* | **0.07** |
| Tele-MAST | 6.40 (1.53) | 3.87 (2.51) | 3.82 (2.15) |  |  | 3.89 (0.36) | 3.2-4.6 | 3.84 (0.35) | 3.1-4.5 |
| Standard care | 6.60 (1.74) | 4.40 (2.06) | 5.11 (2.34) |  |  | 4.38 (0.36) | 3.7-5.1 | 5.08 (0.35) | 4.4-5.8 |
| DASS-Depression | *t* = -0.75, *p* = .457 |  |  | Verbal reasoning | *F* = 11.67** | *F* = 7.93** | **0.09** | *F* = 9.03** | **0.10** |
| Tele-MAST | 12.25 (8.73) | 8.42 (6.92) | 8.15 (5.33) |  |  | 8.91 (1.07) | 6.8-11.0 | 8.64 (0.99) | 6.7-10.6 |
| Standard care | 13.90 (10.92) | 13.66 (9.53) | 13.34 (8.94) |  |  | 13.17 (1.07) | 11.0-15.3 | 12.85 (0.99) | 10.9-14.8 |
| GAD-7 | *t* = -1.65, *p* = .104 |  |  | Education | *F* = 8.56** | *F* = 4.19* | **0.05** | *F* = 10.40** | **0.12** |
| Tele-MAST | 8.55 (4.97) | 6.02 (3.21) | 5.72 (3.49) |  |  | 6.40 (0.59) | 4.7-7.3 | 6.11 (0.52) | 4.4-7.0 |
| Standard care | 10.48 (5.47) | 8.49 (4.89) | 8.89 (4.48) |  |  | 8.12 (0.59) | 7.2-9.8 | 8.51 (0.52) | 7.6-10.2 |
| FACT-G | *t* = 1.06, *p* =.291 |  |  | Months post-diagnosis | *F* = 6.10** | *F* = 5.55* | **0.07** | *F* = 4.55* | **0.06** |
| Tele-MAST | 65.57 (15.22) | 76.72 (12.91) | 74.16 (12.06) |  |  | 75.52 (1.97) | 71.6-79.4 | 72.97 (1.92) | 69.5-78.6 |
| Standard care | 62.11 (13.85) | 67.73 (16.46) | 65.96 (16.56) |  |  | 68.93 (1.97) | 65.0-72.8 | 67.15 (1.92) | 63.3-71.0 |
| FACT-Physical | *t* = 0.787, *p* = .434 |  |  | Months post-diagnosis | *F* = 5.30* | *F* = 2.12 | **0.03** | *F* = 7.08* | **0.08** |
| Tele-MAST | 17.43 (5.32) | 20.15 (4.46) | 19.50 (4.59) |  |  | 19.81 (0.70) | 18.4-21.2 | 19.16 (0.70) | 17.8-20.6 |
| Standard care | 16.48 (5.47) | 18.02 (5.95) | 16.20 (5.79) |  |  | 18.36 (0.70) | 17.0-19.8 | 16.52 (0.70) | 15.1-17.9 |
| FACT-Social | *t* = -0.76, *p* = .450 |  |  | Relationship status | *F* = 0.50 | *F* = 1.68 | **0.02** | *F* = 0.01 | **0.00** |
| Tele-MAST | 18.02 (6.36) | 20.43 (5.03) | 19.27 (4.88) |  |  | 20.54 (0.68) | 19.2-21.9 | 19.38 (0.68) | 18.1-20.6 |
| Standard care | 19.00 (5.18) | 19.39 (4.95) | 19.54 (4.78) |  |  | 19.29 (0.68) | 17.9-20.6 | 19.43 (0.68) | 18.2-20.7 |
| FACT-Emotional | *t* = 1.50, *p* = .137 |  |  | - | *F* = 10.83** | *F* = 10.64** | **0.12** | *F* = 5.54* | **0.07** |
| Tele-MAST | 14.65 (5.20) | 17.41 (3.16) | 17.48 (2.63) |  |  | 17.13 (0.50) | 16.1-18.1 | 17.19 (0.50) | 16.2-18.2 |
| Standard care | 12.97 (4.81) | 14.51 (4.13) | 15.24 (4.11) |  |  | 14.80 (0.50) | 13.8-15.8 | 15.53 (0.50) | 14.5-16.5 |
| FACT-Functional | *t* = 1.55, *p* = .125 |  |  | - | *F* = 6.60* | *F* = 6.74* | **0.08** | *F* = 4.73* | **0.06** |
| Tele-MAST | 15.45 (5.21) | 18.70 (4.39) | 17.99 (4.11) |  |  | 18.46 (0.65) | 17.2-20.2 | 17.65 (0.68) | 16.5-19.5 |
| Standard care | 13.55 (5.75) | 15.90 (5.19) | 15.64 (5.51) |  |  | 16.20 (0.65) | 14.4-17.4 | 15.69 (0.68) | 14.1-17.2 |
| FACT-Br | *t* = -1.16, *p* = .252 |  |  | Verbal reasoning | *F* = 2.19 | *F* = 3.41 | **0.04** | *F* = 0.36 | **0.00** |
| Tele-MAST | 56.68 (13.56) | 64.02 (14.32) | 60.79 (13.10) |  |  | 63.30 (1.90) | 59.5-67.1 | 60.07 (1.67) | 56.7-63.4 |
| Standard care | 54.48 (11.87) | 57.61 (12.84) | 57.92 (12.26) |  |  | 58.33 (1.90) | 54.5-62.1 | 58.64 (1.67) | 55.3-62.0 |
| MQOL-EW | *t* = 0.673, *p* = .503 |  |  | Recurrence | *F* = 5.71* | *F* = 6.98* | **0.08** | *F* = 2.55 | **0.03** |
| Tele-MAST | 6.58 (1.96) | 7.44 (1.34) | 7.13 (1.43) |  |  | 7.37 (0.21) | 7.0-7.8 | 7.07 (0.22) | 6.6-7.5 |
| Standard care | 6.28 (2.04) | 6.54 (1.68) | 6.52 (1.77) |  |  | 6.61 (0.21) | 6.2-7.0 | 6.58 (0.22) | 6.1-7.0 |

EMM = estimated marginal mean. ^a^ η_p_^2^ = partial eta squared: small = .01; medium = .06; large = .14.

Note: Greater months since diagnosis was associated with higher depression and distress and lower QoL; Those in a relationship had better social QoL; individuals with higher verbal reasoning had better QoL and neurocognitive function; recurrence (Y/N) was associated with lower existential well-being

Table S4. Comparison of Demographic and Clinical Variables for Individuals Meeting the Minimal Clinically Important Difference (MCID) on the MADRS (Improved = change score >6)

|  | Tele-MAST | | | | |  | Standard care | | | | |  |
| --- | --- | --- | --- | --- | --- | --- | --- | --- | --- | --- | --- | --- |
|  | Pre- vs post-intervention | |  | Pre- vs 6-weeks post-intervention | | | Pre- vs post-intervention | |  | Pre- vs 6-weeks post-intervention | | |
|  | Improved  (*n* = 28) *M(SD), N* | No change^a^  (*n* = 12)  *M(SD), N* | *ꭓ*^2^/t | Improved  (*n* = 23) *M(SD), N* | No change^b^  (*n* = 17) *M(SD), N* | *ꭓ*^2^/t | Improved  (*n* = 15)  *M(SD), N* | No change^c^ (*n* = 25)  *M(SD), N* | *ꭓ*^2^/t | Improved  (*n* = 15) *M(SD), N* | No change^d^ (*n* = 25)  *M(SD), N* | *ꭓ*^2^/t |
| Gender, F/M | 16/12 | 6/6 | 0.17 | 11/12 | 11/6 | 1.13 | 9/6 | 18/7 | 0.62 | 9/6 | 18/7 | .62 |
| Relationship:  Y/N | 22/6 | 8/4 | NS^e^ | 18/5 | 12/5 | 0.31 | 10/5 | 17/8 | 0.01 | 11/4 | 16/9 | NS^e^ |
| Tumor type  Benign/LGG/HGG | 10/6/12 | 5/3/4 | NS^e^ | 8/4/11 | 7/5/5 | NS^e^ | 4/4/7 | 9/3/13 | NS^e^ | 5/2/8 | 8/5/12 | NS^e^ |
| Recurrence: Y/N | 6/22 | 1/11 | NS^e^ | 5/18 | 2/15 | NS^e^ | 2/13 | 7/18 | NS^e^ | 1/14 | 8/17 | NS^e^ |
| Age (years) | 48.07 (11.8) | 47.75 (16.2) | 0.07 | 50.43 (11.9) | 44.65 (14.1) | 1.41 | 41.10 (15.9) | 50.36 (14.6) | 1.89 | 46.00 (14.7) | 47.40 (16.3) | 0.27 |
| Education (years) | 14.25 (2.2) | 14.25 (2.3) | 0.00 | 14.63 (2.4) | 13.74 (1.9) | 1.27 | 13.67 (3.3) | 14.52 (3.6) | 0.75 | 15.20 (4.3) | 13.60 (2.8) | 1.44 |
| Time since diagnosis (months) | 41.50 (43.4) | 34.00 (34.4) | 0.53 | 32.22 (37.3) | 48.76 (44.1) | 1.28 | 33.37 (30.9) | 61.04 (83.0) | 1.51 | 37.6 (56.7) | 58.44 (75.5) | 0.92 |
| Global cognitive status | -0.37 (0.9) | -0.43 (0.6) | 0.22 | -0.37 (0.9) | -0.41 (0.7) | 0.17 | -0.54 (0.7) | -0.33 (1.1) | 0.65 | -0.41 (0.8) | -0.41 (1.1) | .00 |
| Verbal reasoning | 9.39 (2.4) | 10.08 (3.2) | 0.76 | 9.00 (2.9) | 10.04 (2.3) | 1.26 | 9.53 (2.0) | 8.88 (3.6) | 0.65 | 9.53 (2.7) | 8.88 (3.3) | 0.65 |
| Initial distress (DT) | 6.43 (1.5) | 6.33 (1.6) | 0.18 | 6.44 (1.6) | 6.35 (1.5) | 0.17 | 7.00 (1.9) | 6.34 (1.6) | 1.13 | 7.27 (1.7) | 6.2 (1.7) | 1.95 |
| Sessions with caregiver | 1.11 (2.5) | 1.58 (3.5) | 0.49 | 1.27 (2.7) | 1.24 (2.9) | 0.03 | - | - | - | - | - | - |
| Number of therapy sessions | 8.89 (2.6) | 8.00 (2.6) | 0.97 | 8.83 (2.8) | 8.35 (2.6) | 0.55 | 3.13 (1.8) | 3.48 (1.6) | 0.63 | 3.53 (1.7) | 3.24 (1.7) | 0.53 |
| Therapy alliance | 9.40 (0.84) | 9.73 (0.55) | 1.25 | 9.38 (0.9) | 9.64 (0.5) | 1.04 | 9.61 (0.8) | 9.59 (0.6) | 0.14 | 9.39 (1.0) | 9.71 (0.7) | 1.09 |

^a^Includes 4 people who declined by >6 on the MADRS in the Tele-MAST condition (pre- vs post-intervention)

^b^Includes 6 people who declined by >6 on the MADRS in the Tele-MAST condition (pre- vs 6-weeks follow-up)

^c^Includes 6 people who declined by >6 on the MADRS in the standard care condition (pre- vs post-intervention)

^d^Includes 4 people who declined by >6 on the MADRS in the standard care condition (pre- vs 6-weeks follow-up)

^e^Value not reported for Fisher’s Exact Test (not significant)

Table S5. Baseline Differences and Mixed Model Analysis Comparing Mental Health and Quality of Life for Family Caregivers (*n* = 36) in Each Intervention Across Timepoints, Controlling for Baseline Functioning (Pre-intervention)

| Measure | Pre-intervention (Baseline) | | *t* | Post-intervention | | 6-weeks follow-up | | *F* | *p* |
| --- | --- | --- | --- | --- | --- | --- | --- | --- | --- |
|  | Tele-MAST  *M(SD)* | Standard care  *M(SD)* |  | Tele-MAST  *EMM^a^ (SE)* | Standard care  *EMM^a^ (SE)* | Tele-MAST  *EMM^a^ (SE)* | Standard care  *EMM^a^ (SE)* |  |  |
| DASS-21 |  |  |  |  |  |  |  |  |  |
| Depression | 5.78 (6.25) | 12.78 (10.04) | 2.51* | 7.18 (1.22) | 10.26 (1.22) | 10.00 (1.86) | 12.98 (1.86) | 2.60 | .116 |
| Anxiety | 4.33 (4.67) | 11.00 (8.84) | 2.82** | 7.53 (1.06) | 6.13 (1.06) | 6.58 (1.44) | 8.79 (1.44) | 0.06 | .802 |
| Stress | 15.33 (8.57) | 20.78 (8.21) | 1.95 | 15.76 (1.80) | 17.23 (1.80) | 16.29 (2.50) | 18.67 (2.50) | 0.47 | .499 |
| WHO-QOL |  |  |  |  |  |  |  |  |  |
| Physical | 55.60 (13.90) | 50.33 (14.13) | 1.13 | 56.67 (1.40) | 54.61 (1.40) | 54.17 (2.22) | 51.12 (2.22) | 1.30 | .262 |
| Psychological | 71.18 (15.34) | 56.44 (18.84) | 2.57** | 61.85 (2.31) | 60.39 (2.31) | 59.80 (2.71) | 54.28 (2.71) | 1.07 | .309 |
| Social | 66.33 (18.69) | 59.18 (21.50) | 1.07 | 65.05 (2.53) | 64.76 (2.53) | 57.27 (3.06) | 54.49 (3.06) | 0.19 | .662 |
| Environmental | 77.10 (16.34) | 70.85 (16.30) | 1.15 | 76.25 (2.10) | 78.54 (2.10) | 85.36 (0.94) | 87.25 (0.94) | 1.34 | .255 |

^a^Estimated marginal means adjusting for baseline functioning

*p** < .05, *p*** < .01

Table S6: Within-subjects Comparison of Mental Health and Quality of Life between Pre-intervention and 6-months Post-Intervention for Participants Completing the Tele-MAST Intervention (*n* = 42)

| Measure | Pre-intervention  *M(SD)* | 6-Months Post-Intervention  *M(SD)* | *t* (Cohen’s *d*) |
| --- | --- | --- | --- |
| MADRS | 19.69 (7.86) | 12.60 (7.42) | 5.89*** (0.91) |
| DASS-Depression | 11.95 (9.90) | 8.38 (7.49) | 2.37* (0.37) |
| GAD-7 | 8.62 (4.67) | 6.53 (3.89) | 2.91** (.45) |
| DT | 6.56 (1.57) | 4.17 (2.45) | 5.84*** (0.90) |
| FACT-G | 65.61 (13.62) | 74.31 (17.45) | -3.81*** (0.60) |
| FACT-Physical well-being | 17.76 (5.09) | 19.63 (5.99) | -2.23* |
| FACT-Social QoL | 18.84 (5.35) | 19.77 (5.61) | -1.17 (0.18) |
| FACT-Emotional QoL | 14.58 (4.76) | 17.07 (3.98) | -3.69*** (0.58) |
| FACT-Functional QoL | 14.32 (5.44) | 18.07 (5.76) | -4.49*** |
| FACT-Br | 55.23 (11.83) | 60.03 (16.07) | -2.35* (0.37) |
| MQOL-Existential well-being | 6.31 (2.05) | 7.47 (1.82) | -3.55** (0.55) |

**p*<.05, ***p*<.01, ****p*<.001
